# A Glycolipid Adjuvant, 7DW8-5, Enhances CD8+ T Cell Responses Induced by an Adenovirus-Vectored Malaria Vaccine in Non-Human Primates 

**DOI:** 10.1371/journal.pone.0078407

**Published:** 2013-10-25

**Authors:** Neal N. Padte, Mar Boente-Carrera, Chasity D. Andrews, Jenny McManus, Brooke F. Grasperge, Agegnehu Gettie, Jordana G. Coelho-dos-Reis, Xiangming Li, Douglass Wu, Joseph T. Bruder, Martha Sedegah, Noelle Patterson, Thomas L. Richie, Chi-Huey Wong, David D. Ho, Sandhya Vasan, Moriya Tsuji

**Affiliations:** 1 Aaron Diamond AIDS Research Center, Affiliate of The Rockefeller University, New York, New York, United States of America; 2 Tulane National Primate Research Center, Tulane University Medical Center, Covington, Louisiana, United States of America; 3 Department of Chemistry, the Scripps Research Institute, La Jolla, California, United States of America; 4 Research, GenVec, Inc., Gaithersburg, Maryland, United States of America; 5 US Military Malaria Vaccine Program, Naval Medical Research Center, Silver Spring, Maryland, United States of America; 6 Henry M. Jackson Foundation for the Advancement of Military Medicine, Bethesda, Maryland, United States of America; Federal University of São Paulo, Brazil

## Abstract

A key strategy to a successful vaccine against malaria is to identify and develop new adjuvants that can enhance T-cell responses and improve protective immunity. Upon co-administration with a rodent malaria vaccine in mice, 7DW8-5, a recently identified novel analog of α-galactosylceramide (α-GalCer), enhances the level of malaria-specific protective immune responses more strongly than the parent compound. In this study, we sought to determine whether 7DW8-5 could provide a similar potent adjuvant effect on a candidate human malaria vaccine in the more relevant non-human primate (NHP) model, prior to committing to clinical development. The candidate human malaria vaccine, AdPfCA (NMRC-M3V-Ad-PfCA), consists of two non-replicating recombinant adenoviral (Ad) vectors, one expressing the circumsporozoite protein (CSP) and another expressing the apical membrane antigen-1 (AMA1) of *Plasmodium falciparum*. In several phase 1 clinical trials, AdPfCA was well tolerated and demonstrated immunogenicity for both humoral and cell-mediated responses. In the study described herein, 25 rhesus macaques received prime and boost intramuscular (IM) immunizations of AdPfCA alone or with an ascending dose of 7DW8-5. Our results indicate that 7DW8-5 is safe and well-tolerated and provides a significant enhancement (up to 9-fold) in malaria-specific CD8+ T-cell responses after both priming and boosting phases, supporting further clinical development.

## Introduction

The eradication of global pathogens responsible for endemic and pandemic diseases hinges upon the development of effective vaccines, yet a successful vaccination approach against one of the world’s most prevalent global infectious diseases, malaria, has remained elusive. Several malaria subunit vaccine candidates have demonstrated weak to modest efficacy in early phase clinical trials by delaying the onset of parasitemia or clinical illness in a limited number of subjects [1–3], which suggests that a stronger immune response to these vaccine candidates may afford true sterile protection. There have been many attempts toward the discovery of adjuvants that can enhance the efficacy of existing vaccines not only against malaria but also other infectious diseases and cancers, including adjuvants that target pattern recognition receptors (PRRs) expressed on antigen-presenting cells (APCs) [4–7]. However, while the promising adjuvants developed to date preferentially enhance the humoral response, no clinical adjuvants to date preferentially enhance the T-cell response. Furthermore, while most adjuvants are effective primarily with protein-based vaccines, there has been no adjuvant developed that can enhance the immunogenicity or the protective efficacy of viral-vectored vaccines. The need for such an adjuvant is underscored by the many studies of malaria, HIV and TB and similar intracellular pathogens, demonstrating that protective immunity is mediated in part by T cells, particularly CD8+ T cells [8–13] and that viral-vectored vaccines have been shown to be potent inducers of the CD8+ T-cell response mediating protection [14–16]. 

Glycolipids (for example, α-galactosylceramide (α-GalCer)) bind the non-polymorphic MHC class I-like molecule, CD1d [17,18] and are presented to invariant natural killer T (iNKT) cells, thereby activating *i*NKT cells to rapidly produce large quantities of Th1 and Th2 cytokines, and subsequently induce the activation of a cascade of immuno-competent cells, including dendritic cells (DCs), natural killer (NK) cells, B cells, and CD4+ and CD8+ T cells [18]. α-GalCer can therefore be utilized as a potential direct therapy for cancer and autoimmune and infectious diseases [19–29]. α-GalCer has also been used as an adjuvant to enhance the efficacy of various existing or new vaccines, including malaria vaccines [30–34]. The adjuvant effect of α-GalCer is shown to be mediated by CD1d molecules, *i*NKT cells, IFN-γ, type I interferon, and CD40-CD40L interaction [30,31,33]. We have recently identified 7DW8-5 as the most biologically potent glycolipid among the 25 α-GalCer analogs tested and found that it has a much stronger bioactivity toward *i*NKT cells and CD1d-bearing DCs as compared to α-GalCer [35]. This analog differs from α-GalCer in that it possesses a fluorinated benzene ring at the end of C10 length fatty acyl chain [35]. Most importantly, when co-administered with a recombinant adenovirus (Ad)-expressing the circumsporozoite (CS) protein of a rodent malaria parasite, *Plasmodium yoelii*, AdPyCS, 7DW8-5 exhibited a significantly stronger adjuvant effect than α-GalCer resulting in the enhancement of both the immunogenicity and protective efficacy of the vaccine in mice [35]. 

While CD1d molecules and *i*NKT cells are relatively conserved between mouse and human [36], the frequency and specificity of *i*NKT cells in NHPs, such as rhesus macaques, are even more similar to those in human [37–40]. Therefore, in this study, we sought to test the biological activities and adjuvant effect of 7DW8-5 in the more relevant NHP model, using the same clinically tested human malaria vaccine candidate, AdPfCA, as in our murine studies, prior to commitment to clinical development [41]. AdPfCA (NMRC-M3V-Ad-PfCA; Naval Medical Research Center + Multi-stage Multi-antigen Malaria Vaccine + Adenovirus serotype 5-vectored + *P. falciparum* CSP and AMA1) is a cocktail of two replication-deficient, recombinant Ad serotype 5 vectors, encoding for the sporozoite/early hepatic stage antigen, circumsporozoite protein (*Pf*CSP), or the sporozoite/hepatic stage/erythrocytic stage antigen, apical membrane antigen-1 (*Pf*AMA1) of *P. falciparum* (3D7 strain). In several phase 1 clinical trials in malaria-naïve adult volunteers, AdPfCA was well tolerated and produced strong *Pf*CSP- and *Pf*AMA1-mediated T-cell responses and modest humoral responses [42,43]. Despite the relatively high cell-mediated immune responses to AdPfCA, volunteers later challenged by the bite of *P. falciparum*-infected mosquitoes all became parasitemic [43].

## Materials and Methods

### Ethics Statement

All rhesus macaques (*Macaca mulatta*) were born and maintained at the Tulane National Primate Research Center (TNPRC) in accordance with the regulations of the Committee on the Care and Use of Laboratory Animal Resources. TNPRC is fully accredited by the Association for Assessment and Accreditation of Laboratory Animal Care International. All work conducted on the animals used in this study was performed under protocols approved by the Tulane University Institutional Animal Care and Use Committee (IACUC). Briefly, the rhesus monkeys used in this study were pair-housed (within treatment groups) in commercial stainless steel primate caging within an Animal Biosafety Level 2 facility. The monkeys' daily diet consisted of a commercial primate diet (Fiber Plus Primate, Purina Mills, St. Louis, MO) with supplemental fresh fruits and vegetables and other food enrichment. Fresh drinking water was provided ad libitum via an automatic watering system. All husbandry, environmental enrichment, veterinary, and other such procedures were performed in compliance with the Guide for the Care and Use of Laboratory Animals and the Animal Welfare Act, and the animal health was monitored daily by the animal care staff and veterinary personnel, available 24/7. Monkeys showing signs of disease or distress that could not be alleviated using standard analgesics and/or chemotherapy were humanely euthanized using an overdose of barbiturates according to the guidelines of the American Veterinary Medical Association. It is noteworthy, however, that none of the animal became ill and/or met the criteria for the IACUC approved endpoint policy during this study period, and, therefore, none of them was euthanized.

### Macaque study design

Twenty-five male Indian-origin rhesus macaques (n = 5 per group) were immunized intramuscularly (IM) with 2 ×10^10^ virus particle units (pu) of AdPfCA, which is a 1:1 mixture of two non-replicating recombinant adenoviral (Ad) vectors, one expressing the *P. falciparum* circumsporozoite protein (*Pf*CSP) and another expressing the *P. falciparum* apical membrane antigen-1 (*Pf*AMA1) (both 3D7 strain), in combination with an ascending dose of 7DW8-5 (0 μg, 0.1 μg, 1 μg, 10 μg or 100 μg) at week 0 (prime) and week 25 (boost) (Figure S1). Both vectors are derived from human serotype 5 and are E1, E4 and E3 (partially) deleted. Both vaccine and adjuvant were manufactured and qualified under GMP conditions at GenVec, Inc., Gaithersburg, MD, and Aptuit, Kansas City, MO, respectively, and the two products were mixed immediately before injection. Each immunization was divided into two 1 mL injections administered IM into the right and left arms (deltoid muscles). All animals were monitored for local reactogenicity and systemic reactogenicity. Blood was drawn at several time points pre- and post- prime and boost for immunological and safety assessments as described below. 

### Isolation of PBMCs from whole blood

Whole blood was collected using EDTA tubes and processed immediately or shipped at room temperature overnight before processing. PBMCs (peripheral blood mononuclear cells) were isolated by Histopaque (Sigma, St. Louis, MO) density gradient separation within 6 hours of blood collection. PBMCs were washed with HBSS (Sigma) and R-10 media (RPMI-1640 with 10% HI-FCS (heat-inactivated fetal calf serum), 2 mM L-glutamine, 1 mM sodium pyruvate, 100 U/mL penicillin, 100 µg/mL streptomycin, and 10 mM HEPES (Sigma)), counted, and either frozen or used fresh for the immunological assays described below.

### IFN-γ ELISpot assay

**Figure 1:**
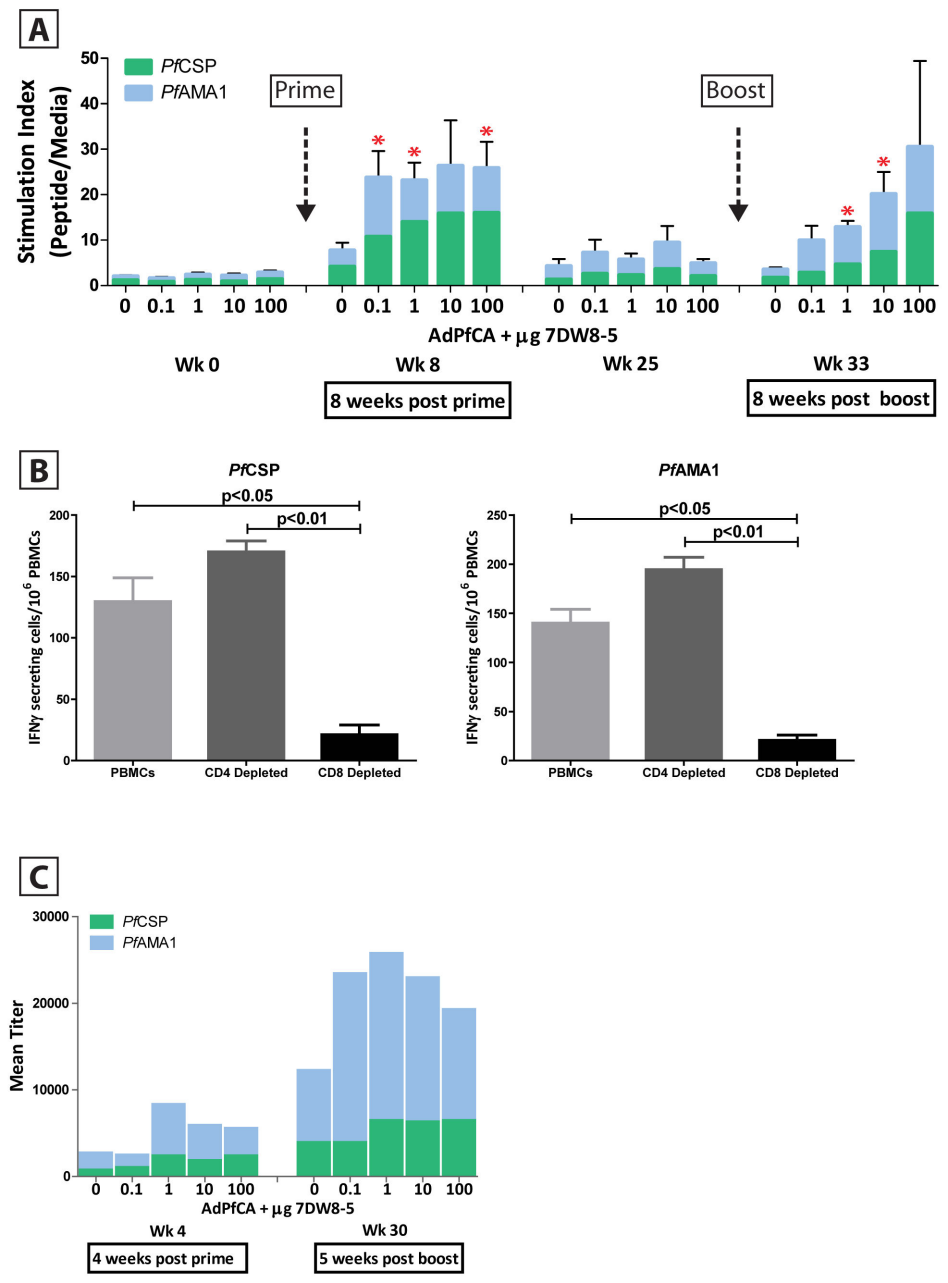
Adjuvant effect of 7DW8-5 on AdPfCA in rhesus macaques. (A) Cellular immunogenicity upon 7DW8-5 and AdPfCA co-administration *in* *vivo*. Five animals per group were vaccinated with AdPfCA alone (control) or in combination with one of four ascending doses of 7DW8-5. PBMCs were isolated before prime and boost and at eight weeks post prime and post boost, stimulated with *Pf*CSP- or *Pf*AMA1-specific peptides, and the number of IFN-γ-secreting cells was measured by ELISpot assay. Stimulation index was calculated as the number of spots detected in the respective peptide stimulated well divided by the number of spots in the media only well, and the bars displayed are the mean of 5 animals per dose group. All samples were run in duplicate. Asterisks represent statistical significance (p < 0.05) for the summation of *Pf*CSP- and *Pf*AMA1 T-cell responses for each respective dose group as compared to the control dose group (AdPfCA + 0 μg 7DW8-5), and error bars represent the standard error for the five animals per group. (B) Enhancement of malaria-specific CD8+ T-cell responses by 7DW8-5. PBMCs from four macaques that received AdPfCA + 100 µg 7DW8-5 were isolated at eight weeks post boost, depleted of CD4+ or CD8+ T cells, stimulated with *Pf*CSP-or *Pf*AMA1-specific peptides, and the relative number of IFN-γ secreting cells were determined by ELISpot assay. All samples were run in duplicate and subtracted for background levels measured in cells stimulated with culture medium containing 0.01% DMSO (negative control). Error bars represent the standard error between duplicated wells. Representative data from one animal are shown. (C) Humoral immunogenicity upon in vivo co-administration of 7DW8-5 and AdPfCA. Animals were vaccinated with AdPfCA alone or in combination with 7DW8-5 as described above. Sera were isolated at four weeks post prime and three weeks post boost and measured for levels of PfCSP- and PfAMA1-directed antibodies. Values presented are a mean of 5 animals per group at each respective time point. All samples were run in duplicate.

ELISpot assays were performed using frozen PBMCs isolated immediately before prime (week 0) and boost (week 25) and at weeks 4, 8, 28, 30 and 33. PBMCs (2.5 × 10^5^ /100 µL) were stimulated in the presence of *Pf*CSP and *Pf*AMA1 peptide pools (1 μg/mL), provided by the Naval Medical Research Center (identical to those used in prior clinical trials). R-10 was used as a negative control and SEB (Staphylococcal enterotoxin B; 2 μg/mL, Sigma) was used as a positive control for each animal. The IFN-γ ELISpot assay was performed as per the manufacturer’s instructions (Mabtech, Nacka Strand, Sweden) and IFN-γ-producing cells were counted manually. Because of the considerable varying degree of background responses (anywhere between 20 spots and 400 spots per million PBMCs in the absence of the peptides) observed in each macaque sample, the net spot counts subtracted by the background spot counts could not accurately represent the degree of T cell response. Therefore, we calculated stimulation indices, the number of spots detected in the respective peptide stimulated well divided by the number of spots in the media only well, in order to evaluate T cell response. For ELISpot assays that present data from an individual rhesus macaque (Figure 1B and Figure S2A), PBMCs were stimulated in the presence of *Pf*CSP and *Pf*AMA1 peptide pools (Figure 1B) or in the presence of glycolipid (Figure S2A), and the T-cell response was calculated as the net spot counts subtracted by the background spot counts.

The CD4+ and CD8+ T cell depletions were performed using frozen PBMCs isolated from macaques at week 33 (8 weeks after boosting). Briefly, Dynabeads were conjugated as per the manufacturer’s instructions (Invitrogen Dynal AS, Oslo, Norway) with purified mouse anti-human CD4 clone L200 or with purified mouse anti-human CD8 clone RPA-T8 (BD Biosciences, San Jose, CA) to deplete PBMCs of CD4+ and CD8+ T cells, respectively. Depletion of the indicated cell population was confirmed by cell staining and flow cytometry and IFN-γ ELISpot assays were performed on the purified cells as described above. 

### ELISA

Whole blood was collected using clot tubes and shipped at room temperature overnight for processing. Serum was isolated from clotted blood within 24 hours of blood collection. *Pf*CSP- and *Pf*AMA1-directed antibodies were measured by ELISA from samples collected immediately before prime (week 0) and boost (week 25) and at weeks 4, 28, and 30. 96-well plates were coated with 0.2 M carbonate-bicarbonate buffer (ThermoFisherScientific, Waltham, MA) containing *Pf*CSP (250 ng/mL) or *Pf*AMA1 (500 ng/mL) protein at 4°C overnight. Plates were then washed three times with PBS containing 0.05% Tween-20 (Sigma) and blocked with ELISA Assay Diluent (BioLegend, San Diego, CA) for 2 hours. Samples were serially diluted in blocking solution, added to the ELISA plate in duplicates and incubated at 4°C overnight. Plates were washed and incubated with mouse anti-monkey IgG-HRP (SouthernBiotech, Birmingham, AL). Plates were developed with TMB substrate solution (Sigma), the reaction was stopped with 2N H_2_SO_4_, and the OD at 450nm was measured using an ELISA reader (Dynex Technologies, Chantilly, VA). The end-point antibody titers were calculated as the reciprocal of the last dilution that was at least 2-fold higher than baseline sera (sera collected before prime) and yielded an OD ≥ 0.1.

### Cell staining

Fresh PBMCs, isolated on day 0 before vaccination (baseline) and days 1, 2, 3, 7 and 14 post prime, were stained for NK cells, *i*NKT cells, DCs and DC activation markers. One million PBMCs per condition were resuspended in 250 μL of FACS buffer (PBS + 1% HI-FCS), washed and stained for 30 minutes at 4°C with CD3 APC, CD56 PerCP-Cy 5.5 (BD Biosciences), Vα24-Jα18 (6B11) PE (BioLegend, San Diego, CA) and, Live/Dead blue (Invitrogen, Carlsbad, CA) for the identification of NK and *i*NKT cells. DCs were identified by staining with CD14 PerCP-Cy 5.5, CD16 PerCP-Cy 5.5, CD3 V450, CD56 V450, CD19 V450, CD80 APC-H7, CD86 Alexa700, CD40 APC (BD Biosciences), CD11c PE (BioLegend) and Live/Dead blue. The cells were washed twice and fixed with 25% Cytofix (BD Biosciences) in PBS prior to FACS analysis. Samples were acquired using a BD LSRII, and all data was analyzed using FACS DIVA software (BD Biosciences). CD3 and CD56 were used to determine the percentage of NK cells (CD3-CD56+) and CD3 and Vα24-Jα18 were used to identify the percentage of *i*NKT cells (CD3+ Vα24-Jα18+). To isolate DCs, cells were first gated on CD3-/CD56-/CD19- and CD14+/CD16+ populations, and then gated on CD11c+/CD14^high^ myeloid DC populations, and lastly activated DCs were identified by further gating on CD40+, CD80+ and CD86+ populations. 

### Serum cytokines

Serum concentrations of the cytokines GM-CSF, IL-2, IL-6, IL-8, IL-12p70 and IL-1β were measured at baseline and at 2, 4, 6, 8, 12, 18 and 24 hours after priming using the Human ProInflammatory 9-Plex Assay Ultra-Sensitive Kit as per the manufacturer’s instructions (Mesoscale, Rockville, MD). Due to the lack of cross reactivity between the human kit and rhesus IFN-γ, IFN-γ serum levels were measured with the Monkey IFN-γ ELISA kit as per the manufacturer’s instructions (Cell Sciences, Canton, MA) at the same time points as in the multiplex assay.

## Results

### Biological activity of 7DW8-5 against rhesus macaque PBMCs in vitro

In order to verify the species relevancy of rhesus macaques for our study, we first sought to determine the specificity and stimulatory activity of 7DW8-5 *in vitro* against PBMCs derived from the animals selected for inclusion in the study. As shown in Figure S2A, 7DW8-5 displayed a potent stimulatory activity, resulting in IFN-γ secretion by a higher number of PBMCs compared to α-GalCer at all concentrations. Because the percentage of *i*NKT cells in rhesus macaques is highly variable, similar to humans, we distributed these animals among the five test groups so that the mean percentage of *i*NKT cells was similar across groups (Figure S2B). 

### Adjuvant activity of 7DW8-5 on CD8+ T-cell responses induced by AdPfCA in rhesus macaques

25 male Indian-origin rhesus macaques (n = 5 per group) were immunized intramuscularly (IM) with 2 ×10^10^ virus particle units (pu) of AdPfCA, the optimal adenovirus dose identified for malaria-naïve adults in previous phase 1 clinical trials, in combination with an escalating dose of 7DW8-5 (0 μg, 0.1 μg, 1 μg, 10 μg or 100 μg) in a prime-boost vaccination regimen (Figure S1). Each immunization was divided into two 1 mL shots that were administered to the right and left arms (deltoid muscles) because dividing the dose between distinct sites has been shown in smaller animal models to increase immunogenicity [44]. The lowest adjuvant dose tested (0.1 μg) corresponded roughly to the optimal dose in mice, while the highest adjuvant dose tested (100 μg) corresponded roughly to the equivalent human dose calculated on a weight for weight basis.

In order to determine whether or not 7DW8-5 could display an adjuvant effect on AdPfCA in rhesus macaques, PBMCs and sera were collected and analyzed at various time points post prime and boost. We first determined the level of malaria-specific T-cell response by an ELISpot assay. For this purpose, frozen PBMCs were thawed and incubated with 15 amino acid (aa) synthetic peptides overlapping by 11 aa covering the entire *Pf*CSP- and *Pf*AMA1 antigens and the relative numbers of IFN-γ secreting T cells were determined. We found that 7DW8-5 potently enhances the T-cell response to both *Pf*CSP and *Pf*AMA1 antigens upon both priming and boosting phases (Figure 1A). All doses of 7DW8-5 demonstrated potent activity, with some enhancing the total T-cell response by over 9-fold when compared to the control arm (AdPfCA alone). While the enhancement of T-cell response appears to be irrespective of the amount of 7DW8-5 given during the first immunization, a clear dose-response relationship is observed after a second immunization, with the highest adjuvant dose group (100 μg 7DW8-5) being the only one to reproducibly maintain the high level of cellular enhancement observed after the first immunization. Importantly, the magnitude of the enhancement of T-cell response by 7DW8-5 was irrespective of circulating *i*NKT cell level (Figure S3), although the possible influence of NKT cell variability at the local tissue level was not assessed, and indicates that this adjuvant may be useful across broad populations. It is noteworthy that the same sets of overlapping peptides used in our ELISpot assay were able to detect more malaria-specific IFN-γ-secreting CD8+ T cells than CD4+ T cells from PBMCs of human volunteers vaccinated with AdPfCA [42,43]. In view of these earlier results observed in AdPfCA-vaccinated humans and the enhanced cellular immunogenicity observed in the NHP reported here, we investigated the type of T-cell response that is enhanced by 7DW8-5. PBMCs from four macaques that received the prime-boost regimen of AdPfCA co-jointly with 100 μg 7DW8-5 were depleted of CD4+ or CD8+ T cells and then stimulated with either *Pf*CSP- and *Pf*AMA1-specific peptides. We found that the number of malaria-specific IFN-γ secreting T cells greatly diminished upon depletion of CD8+ T cells, but not CD4+ T cells, thus indicating that the majority of malaria-specific T-cell responses enhanced by 7DW8-5 in the NHPs were CD8+ T cells (Figure 1B). Next, we determined the level of malaria-specific humoral response in sera by ELISA (Figure 1C). As expected with glycolipid-based adjuvants, we observed a modest, but not significant, enhancement in the humoral immune response. We observed a trend towards a 2- to 3-fold enhancement in the titer of *Pf*CSP-specific antibodies when macaques were primed with AdPfCA formulated with 1 μg, 10 μg or 100 μg of 7DW8-5; the level of *Pf*AMA1-specific humoral response was also increased when AdPfCA was co-administered with 7DW8-5. 

### Activity of 7DW8-5 on the innate immune responses in rhesus macaques

**Figure 2:**
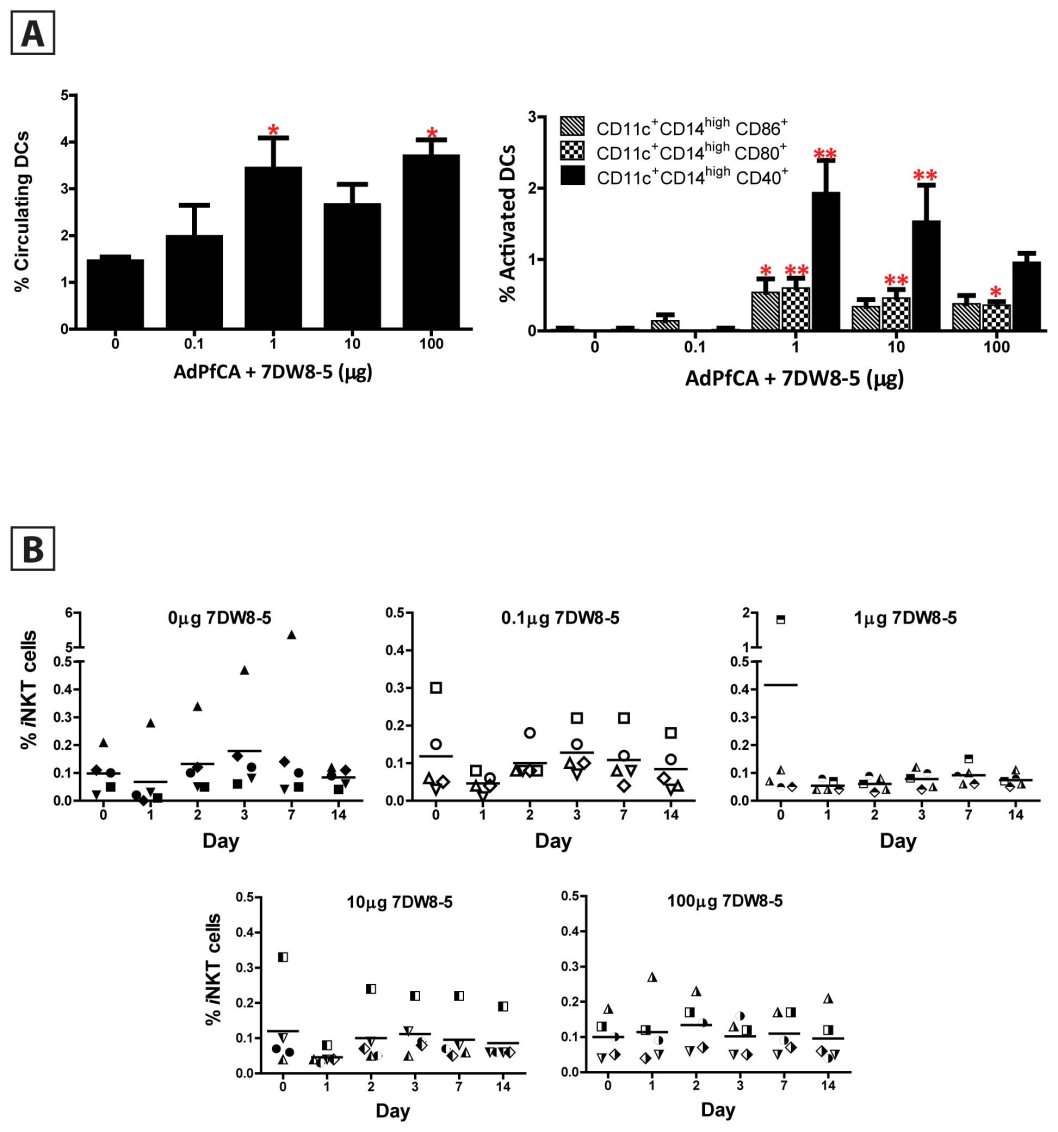
Activity of 7DW8-5 on DCs and *i*NKT cells in rhesus macaques. (**A**) Percentage of circulating and activated monocytoid DCs upon 7DW8-5 and AdPfCA co-administration *in* *vivo*. PBMCs were isolated 24 hours post prime and stained for circulating (left panel) and activated (right panel) DCs. Each column indicates the mean value for 5 animals per group; errors bars denote SEM. * = p < 0.05 and ** = p <0.01 when compared to control group receiving AdPfCA + 0 µg 7DW8-5. (**B**) AdPfCA administration with or without 7DW8-5 induces a transient decrease in the percentage of iNKT cells. PBMCs were isolated at baseline and up to 2 weeks post prime and stained for iNKT cells as described. Each point represents % iNKT cells from one animal; lines indicate mean % iNKT cells per dose group at the indicated time point.

Glycolipids presented by CD1d molecules activate *i*NKT cells, which in turn induce activation and maturation of DCs before subsequent activation of NK cells, B cells, CD4+ and CD8+ T cells. Therefore, to determine if 7DW8-5 enhanced the innate immune response in macaques, we measured the percentage and activation status of DCs upon AdPfCA and 7DW8-5 co-administration (Figure 2A). We opted to measure CD11c+CD14^high^ monocyte-derived (monocytoid) DCs in the peripheral blood of rhesus macaques, as previously described [45]. We found that not only did 7DW8-5 increase the percentage of circulating monocytoid DCs at 1 day post-priming, but it also elicited the activation of monocytoid DCs, as measured by CD40, CD80, and CD86 expression. This phenomenon was most significant when 1 μg, 10 μg and, to a lesser extent, 100 μg of 7DW8-5 was co-administered with AdPfCA. There were no statistically significant differences in the percentages of circulating monocytoid DCs or the activation of monocytoid DCs among the animal groups at baseline before vaccination (data not shown), indicating that the enhancement observed at Day 1 in the groups receiving 1 μg, 10 μg and 100 μg of 7DW8-5 in combination with AdPfCA was due to the presence of 7DW8-5 and not an inherent variability in the number or activation of monocytoid DCs among the different animals (data not shown).

### Activity of 7DW8-5 on the level of iNKT cells in rhesus macaques

Because other glycolipids, such as α-GalCer, cause a decrease in the levels of *i*NKT cells for over a week when administered intravenously (IV) in some clinical trials [46–48], we investigated whether the more potent glycolipid 7DW8-5 also affected *i*NKT cell levels prior to vaccination and on days 1, 2, 3, 7, and 14 after prime (Figure 2B). The number of circulating *i*NKT cells appeared to decrease one day after AdPfCA administration alone and irrespective of AdPfCA co-administration with 0.1, 1, or 10 μg of 7DW8-5. In each of these groups, including the control group, no statistically significant decreases were observed and levels quickly returned to baseline levels by day 2. Interestingly, no decrease in the number of *i*NKT cells was observed after AdPfCA co-administration with 100 μg 7DW8-5, and this was the one dose group that was also able to maintain reproducible levels of cellular enhancement after both prime and boost (Figure 1A). 

### Safety profile of 7DW8-5 in the context of AdPfCA vaccination in rhesus macaques

**Figure 3:**
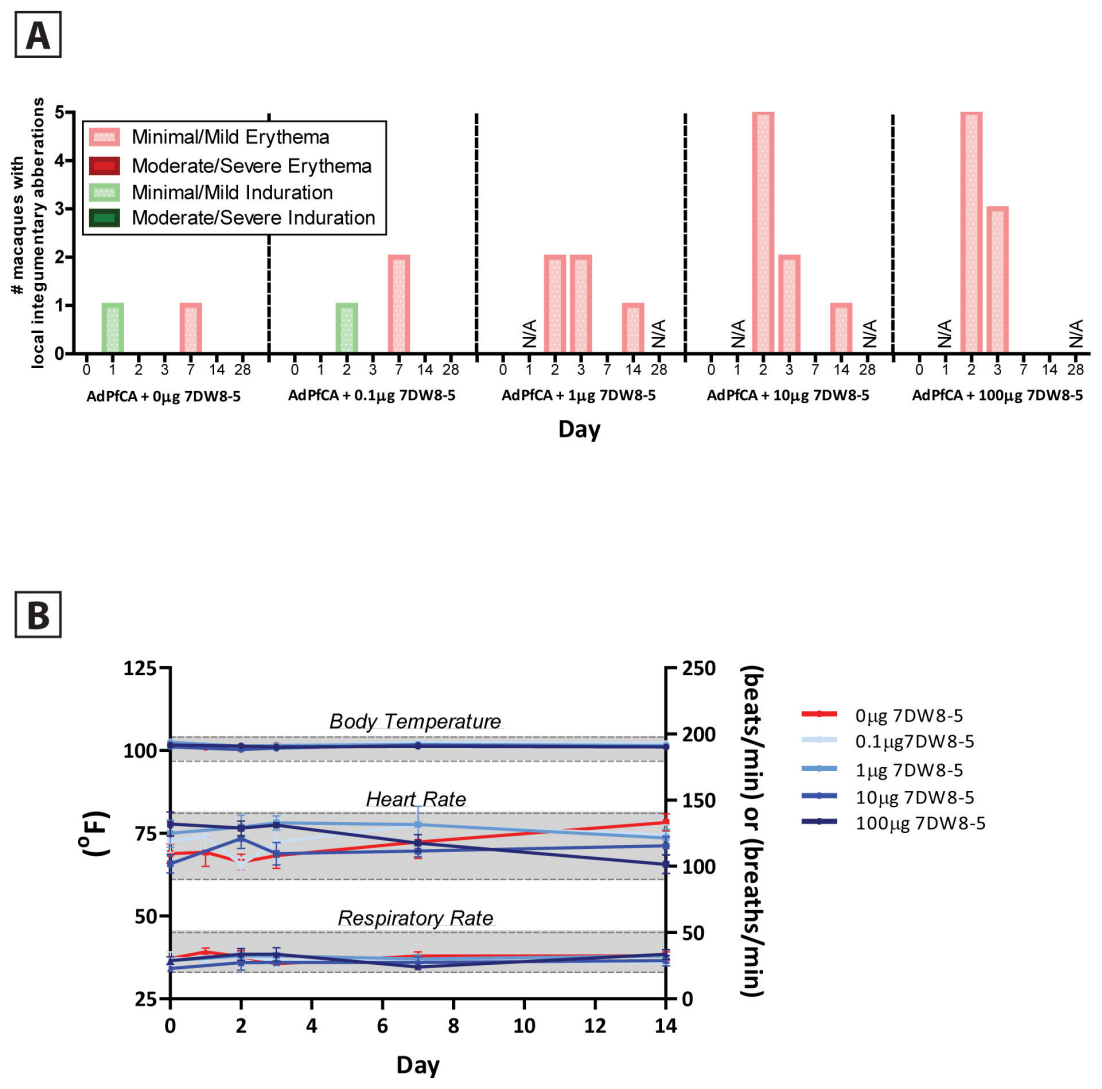
7DW8-5 co-administered with AdPfCA exhibits a favorable safety profile in rhesus macaques. (**A**) Co-administration of 7DW8-5 and AdPfCA induces mild and transient local reactogenicity *in* *vivo*. Animals were vaccinated with AdPfCA alone or in combination with escalating doses of 7DW8-5 and observed for induration and erythema at the vaccination site at baseline and up to 28 days post prime. Minimal/mild classification indicates that the reactogenicity covered <1 cm^2^ of skin surface area. N/A indicates time points where local reactogenicity was not assessed. (**B**) Co-administration of 7DW8-5 and AdPfCA does not induce systemic reactogenicity *in* *vivo*. Animals were monitored for evidence of fever (body temperature, left y-axis), tachycardia (heart rate, right y-axis) and respiratory distress (respiratory rate, right y-axis) at baseline and up to two weeks post prime. Each colored line represents the mean of the indicated 5 animal dose group over time. Error bars indicate SEM, and shaded grey boxes indicate the normal physiological range for each condition.

To assess the safety profile of AdPfCA and 7DW8-5 co-administration in rhesus macaques, we measured local and systemic reactogenicity. Local reactogenicity at the site of injection was monitored on days 0, 1, 2, 3, 7, 14 and 28 post-priming vaccination to evaluate the activity and potential tolerability of the co-administration regimen (Figure 3A). Minimal to mild induration was observed sporadically in one control macaque that received AdPfCA alone and one macaque co-administered the lowest dose of 7DW8-5 (0.1 μg). The number of macaques exhibiting erythema positively correlated with escalating doses of 7DW8-5 co-administered with AdPfCA, however all cases of erythema were mild (covering <1 cm^2^ of skin surface area) and erythema rapidly resolved in all animals after peaking on day 2. These data indicate that the localized erythema was dose dependent and transient. We also determined if 7DW8-5 co-administration with AdPfCA resulted in any systemic reactogenicity by monitoring the macaques for fever, tachycardia and respiratory function both before and up to two weeks after immunization (Figure 3B). Our results indicate that body temperature, heart rate and respiratory rate for all macaques were maintained within normal physiological limits after immunization.

We further assessed safety by characterizing the systemic cytokine profile resulting from immunization. Sera were collected at time 0 and 2, 4, 6, 8, 12, 18 and 24 hours after priming and monitored for any systemic cytokine production, measured by substantial changes in IL-6, IFN-γ, IL-2, IL-12, IL-8, IL-1β, and GM-CSF levels (Figure S4). In the control group (AdPfCA alone with no 7DW8-5), three trends among cytokine expression levels were identified: no change (for IL-2 and IL-12), transient increase (for IL-6 and IFN-γ) or oscillating levels (for IL-8, IL-1β and GM-CSF). These effects were amplified in groups where 7DW8-5 was co-administered, however the highest peak or trough values in the four 7DW8-5 groups were not statistically different when compared to the highest peak or trough values in the control group. Also, no statistically significant changes from baseline values were observed for IFN-γ, IL-2, IL-12, IL-8, IL-1β, and GM-CSF in any dose group 24 hours after vaccination. We observed a slight increase in IL-6 levels 24 hours after vaccination, but this increase was also seen in control rhesus macaques that were vaccinated with AdPfCA alone.

## Discussion

In the current study, we conducted a thorough examination of the immunogenicity, innate immunity and safety of the glycolipid 7DW8-5 in the context of a human malaria vaccine candidate, AdPfCA, in rhesus macaques. Several human trials using the parental compound, α-GalCer, demonstrated the safety of this glycolipid compound [46,49–51]. However, since a biologically more potent analog could potentially cause unanticipated side effects *in vivo*, we sought to investigate both the *in vivo* activity and safety profile of this novel molecule 7DW8-5 in a clinically relevant context, such as an adjuvant for a malaria vaccine candidate. Our current study indicated that 7DW8-5 co-administration with the GMP-manufactured AdPfCA vaccine was safe in NHPs, which have a similar percentage of *i*NKT cells as humans. In addition, with regards to the systemic cytokine production profile, although there was a slight increase in the level of cytokines IL-6 and IFN-γ in the sera of macaques, this increase was observed in the AdPfCA vaccination alone control arm as well. Importantly, the phase 1 safety trials of AdPfCA have indicated no major safety concerns [42,43], suggesting that the transient and slight cytokine increase we observed may not directly translate into an adverse clinical safety profile in humans. We conclude that 7DW8-5 does not induce any significant adverse effects in NHPs when co-injected intramuscularly with an Ad-based malaria vaccine. 

As for the ability of 7DW8-5 to stimulate innate immune responses, 7DW8-5 administration, particularly at 1 μg and 10 μg doses, elicited earlier activation of circulating monocytoid DCs at 1 day post vaccination, as shown by the up-regulation of CD40, CD80, and CD86 expression. It is noteworthy that monocytoid DCs express a low level of CD1d molecules, but the CD1d molecules are able to exhibit an extremely potent glycolipid-presenting function [52]. This corroborates our findings that 7DW8-5 activates monocytoid DCs possibly via stimulation of *i*NKT cells [53]. The administration of a high dose of α-GalCer to animals has been shown to cause a rapid *in vivo* down-regulation of the T-cell receptor of *i*NKT cells, which often lasts for a month and grossly presents as a rapid disappearance of *i*NKT cells due to down modulation of the cell surface receptors typically used to identify these cells [54,55]. However, in contrast to these earlier reports using α-GalCer, 7DW8-5 does not seem to have an apparent effect on the number of *i*NKT cells. This may be in part due to the fact that 7DW8-5 was administered IM in this study, while all the data with α-GalCer was obtained after IV administration. In fact, intranasal or intradermal delivery of α-GalCer has been shown to reduce *i*NKT cell anergy compared with IV administration in mice [56,57].

Overall, our study demonstrates that 7DW8-5 elicits early activation of DCs, a key component of innate immunity, and has a favorable safety profile in rhesus macaques when co-administered with the AdPfCA vaccine. Most strikingly, 7DW8-5 potently enhanced the malaria-specific T-cell response induced by a human malaria vaccine candidate, thereby displaying its potent adjuvant effect. Furthermore, the cellular enhancement by 7DW8-5 was observed not only after priming, but also after boosting that could extend the enhancement up to 33 weeks after priming. This indicates a capacity of 7DW8-5 to induce long lasting malaria-specific T-cell responses. Lastly, our finding via depletion studies that the majority of malaria-specific T-cell responses enhanced by 7DW8-5 are of CD8+ T cells is truly encouraging in light of the fact that, to date, there has been no single adjuvant that can potently enhance a vaccine-induced CD8+ T-cell response or one that can enhance the immunogenicity of viral-vectored vaccines. One of the major goals for a successful malaria vaccine is to elicit a potent CD8+ T-cell response against malaria antigens. 7DW8-5 may therefore be a powerful new tool in the development of vaccines against pathogens requiring a strong cell-mediated immune response, such as malaria, HIV and TB. While it would be ideal to evaluate the efficacy of the 7DW8-5 adjuvant in combination with the AdPfCA vaccine against a malaria challenge in non-human primates, the human malaria parasite, *P. falciparum*, that the AdPfCA vaccine platform is designed to protect against does not infect rhesus macaques. Therefore, the 7DW8-5 adjuvant and AdPfCA vaccine combination would need to be evaluated for efficacy against *P. falciparum* challenge in a future Phase 1 trial with controlled human malaria infection (CHMI). Importantly, for a vaccine that may be administered to healthy individuals, the co-administration of 7DW8-5 and AdPfCA has a favorable safety and immunogenicity profile in NHPs, which have a similar percentage of *i*NKT cells as humans. Our study is the first report investigating the adjuvant effect of a CD1d-binding glycolipid in NHPs, and poises 7DW8-5 to move forward into clinical development.

## Supporting Information

Figure S1 **Study Design.** Rhesus macaques were distributed among one of five dose groups and injected IM with AdPfCA alone (Group 1, control) or with AdPfCA pre-mixed with one of four ascending doses of 7DW8-5 (Groups 2 - 5) in a prime-boost vaccination regimen. Samples were drawn post-prime for safety and innate immunity evaluation and both post-prime and post-boost for cellular and humoral immunogenicity evaluation during the time ranges indicated.
(TIF)

Figure S2 **Rhesus macaque species suitability and randomization.** (A) Activity of 7DW8-5 against rhesus macaque PBMCs in vitro. 1 x 106 PBMCs were isolated from one naïve rhesus macaque and cultured in the presence of escalating doses of 7DW8-5 or α-GalCer in a 96-well Multiscreen-HA plate pre-coated with a captured anti-IFN-γ IgG. After 24 hours of incubation, biotinylated anti-IFN-γ IgG was added to the plate, and the relative number of IFN-γ-secreting cells was determined by an ELISpot assay. All samples were run in duplicate and subtracted for background levels measured in cells stimulated with culture medium containing 0.01% DMSO (negative control). Error bars represent the standard deviation between duplicated wells, and the data represent one of three experiments with similar results, using PBMCs from three different rhesus macaques. (B) Baseline distribution of iNKT cell percentage among rhesus macaques. PBMCs were isolated from all animals pre-vaccination, stained with antibodies against CD3 and Vα24-Jα18, and % iNKT cells were quantified for each animal. Animals were allocated across the five dose groups to ensure that the mean % iNKT cells were equivalent among groups. Each point represents % iNKT cells from one animal; horizontal lines indicate mean % iNKT cells per dose group indicated.
(TIF)

Figure S3 **Adjuvant effect of 7DW8-5 does not correlate with iNKT cell percentage in rhesus macaques.** Analysis of ELISpot stimulation index and percentage of circulating iNKT cells for each group of macaques did not reveal any significant correlation.
(TIF)

Figure S4 **Minimally and transiently increased serum cytokine levels in the sera upon in vivo co-administration of AdPfCA and 7DW8-5.** Serum concentrations of the indicated cytokines were measured at baseline and up to 24 hours post prime in all animals. Changes in IL-2 and IL-12p70 were detectable in only some animals in each dose group. Columns represent the mean values per dose group at the time point indicated and errors bars indicate SEM. Light blue box and dashed lines indicate the range for limit of detection (LOD) for each cytokine measured, which varied slightly among plates. * = p < 0.05 when compared to the 0 hour baseline time point within each dosing group.
(TIF)
