# Supplementary figures and images for: A Glycolipid Adjuvant, 7DW8-5, Enhances CD8+ T Cell Responses Induced by an Adenovirus-Vectored Malaria Vaccine in Non-Human Primates
Source: PLoS One. 2013 Oct 25;8(10):e78407. doi: 10.1371/journal.pone.0078407 (PMC3808339; doi:10.1371/journal.pone.0078407)

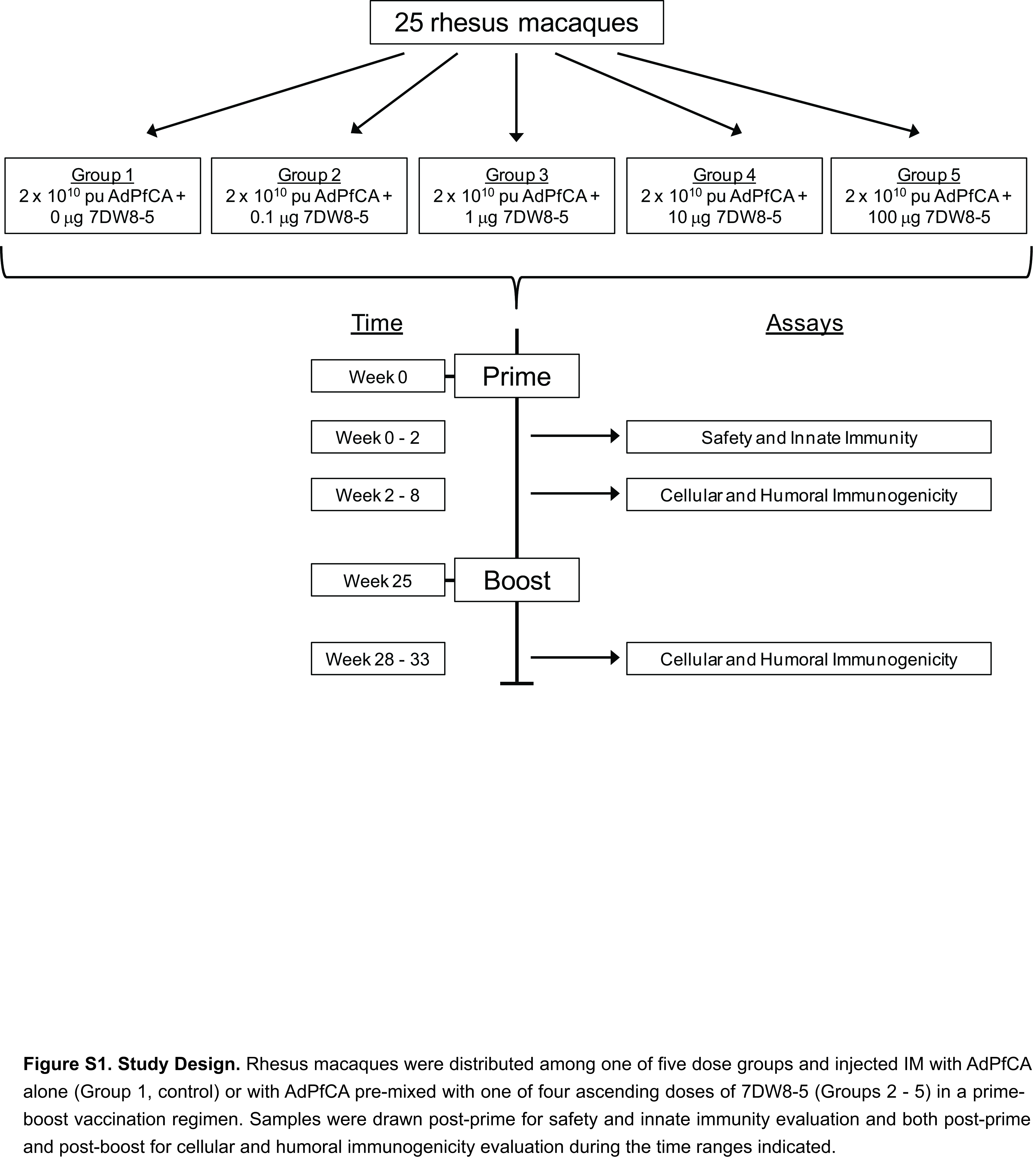

Supplement: Figure S1 — Study Design. Rhesus macaques were distributed among one of five dose groups and injected IM with AdPfCA alone (Group 1, control) or with AdPfCA pre-mixed with one of four ascending doses of 7DW8-5 (Groups 2 - 5) in a prime-boost vaccination regimen. Samples were drawn post-prime for safety and innate immunity evaluation and both post-prime and post-boost for cellular and humoral immunogenicity evaluation during the time ranges indicated. (TIF) [file pone.0078407.s001.tif]

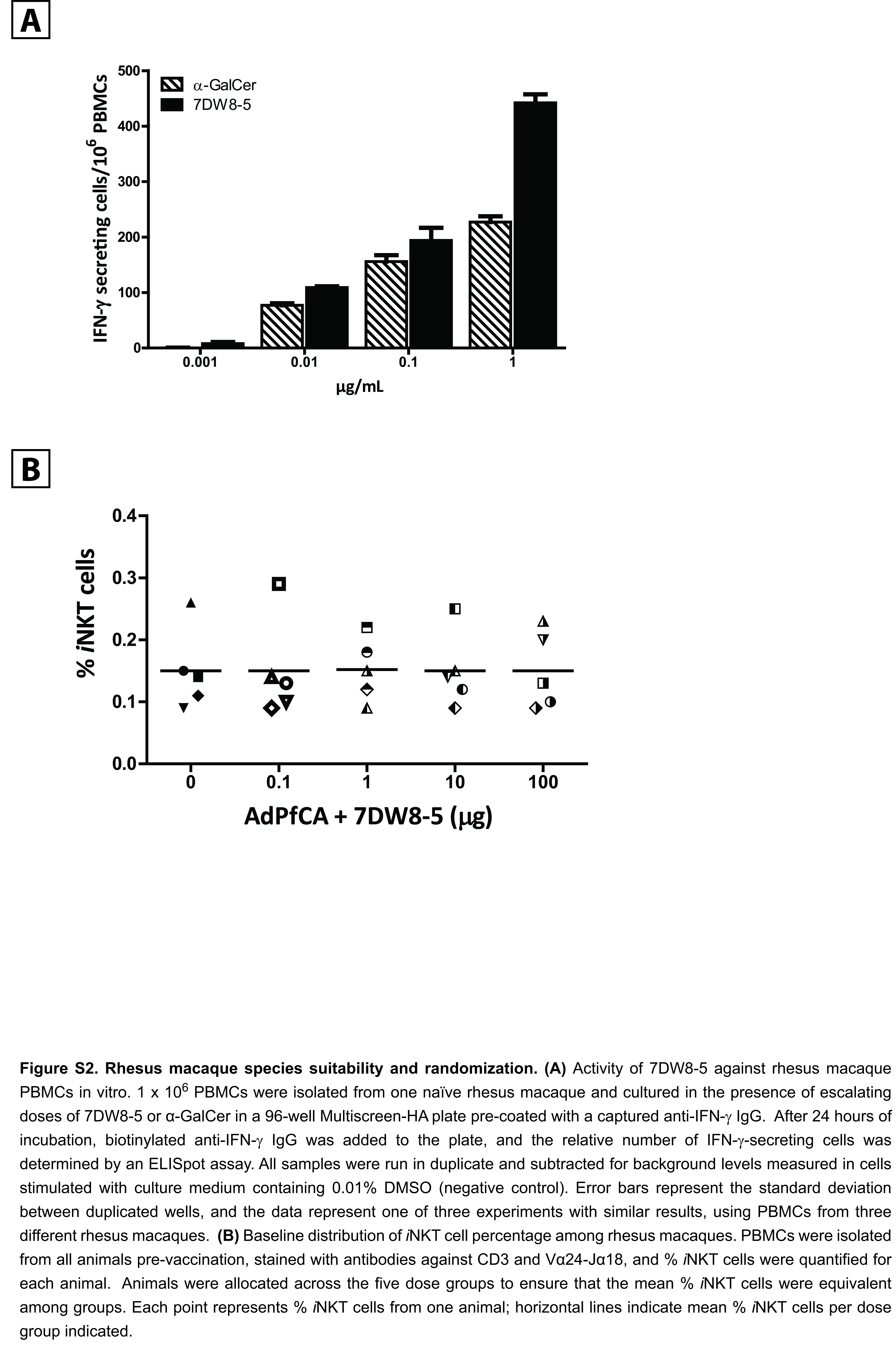

Supplement: Figure S2 — Rhesus macaque species suitability and randomization. (A) Activity of 7DW8-5 against rhesus macaque PBMCs in vitro. 1 x 10^6^ PBMCs were isolated from one naïve rhesus macaque and cultured in the presence of escalating doses of 7DW8-5 or α-GalCer in a 96-well Multiscreen-HA plate pre-coated with a captured anti-IFN-γ IgG. After 24 hours of incubation, biotinylated anti-IFN-γ IgG was added to the plate, and the relative number of IFN-γ-secreting cells was determined by an ELISpot assay. All samples were run in duplicate and subtracted for background levels measured in cells stimulated with culture medium containing 0.01% DMSO (negative control). Error bars represent the standard deviation between duplicated wells, and the data represent one of three experiments with similar results, using PBMCs from three different rhesus macaques. (B) Baseline distribution of iNKT cell percentage among rhesus macaques. PBMCs were isolated from all animals pre-vaccination, stained with antibodies against CD3 and Vα24-Jα18, and % iNKT cells were quantified for each animal. Animals were allocated across the five dose groups to ensure that the mean % iNKT cells were equivalent among groups. Each point represents % iNKT cells from one animal; horizontal lines indicate mean % iNKT cells per dose group indicated. (TIF) [file pone.0078407.s002.tif]

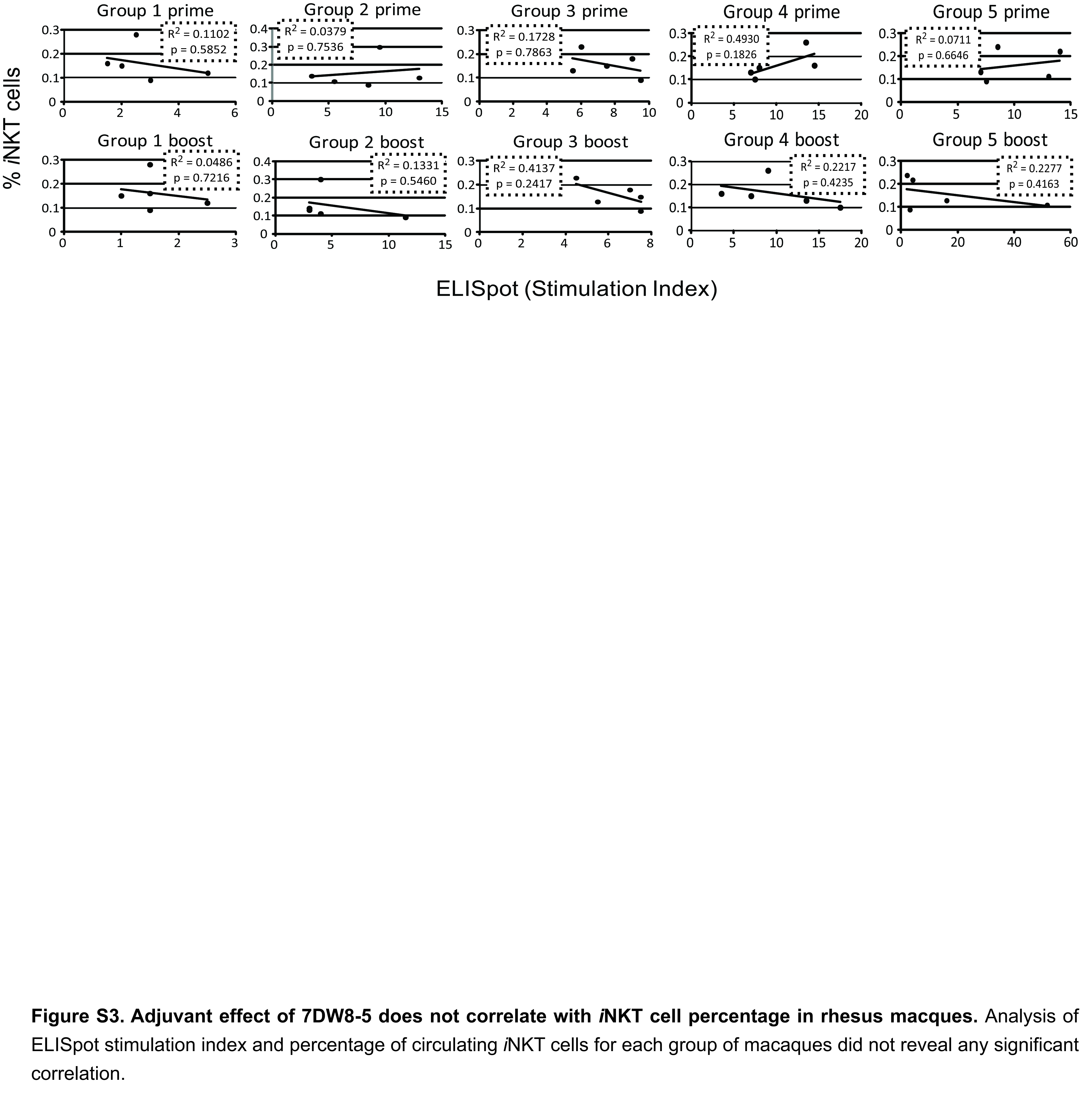

Supplement: Figure S3 — Adjuvant effect of 7DW8-5 does not correlate with iNKT cell percentage in rhesus macaques. Analysis of ELISpot stimulation index and percentage of circulating iNKT cells for each group of macaques did not reveal any significant correlation. (TIF) [file pone.0078407.s003.tif]

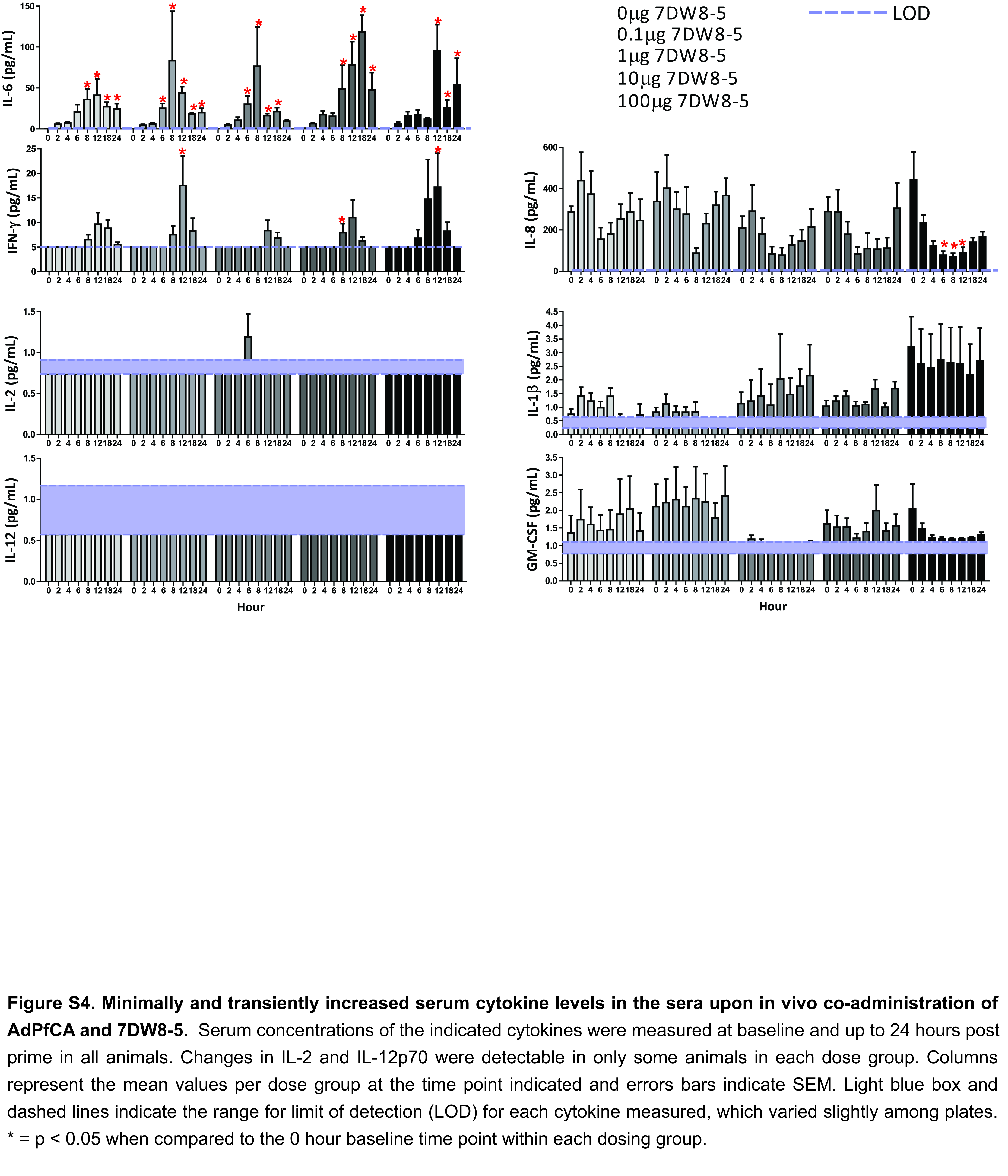

Supplement: Figure S4 — Minimally and transiently increased serum cytokine levels in the sera upon in vivo co-administration of AdPfCA and 7DW8-5. Serum concentrations of the indicated cytokines were measured at baseline and up to 24 hours post prime in all animals. Changes in IL-2 and IL-12p70 were detectable in only some animals in each dose group. Columns represent the mean values per dose group at the time point indicated and errors bars indicate SEM. Light blue box and dashed lines indicate the range for limit of detection (LOD) for each cytokine measured, which varied slightly among plates. * = p < 0.05 when compared to the 0 hour baseline time point within each dosing group. (TIF) [file pone.0078407.s004.tif]
